# Suture granuloma after orchiectomy: sonography, doppler and elastography features

**DOI:** 10.1590/S1677-5538.IBJU.2013.0207

**Published:** 2015

**Authors:** Mustafa Secil, Ugur Mungan, Kutsal Yorukoglu

**Affiliations:** 1 Dokuz Eylul University Faculty of Medicine – Radiology, Inciralti, Izmir, Turkey; 2 Dokuz Eylul University Faculty of Medicine – Urology, Inciralti, Izmir, Turkey; 3 Dokuz Eylul University Faculty of Medicine – Pathology, Inciralti, Izmir, Turkey

## Abstract

Suture granuloma is a mass forming benign lesion that develops at the site of surgery as a foreign body reaction to non-absorbable suture material. We present a case of suture granuloma that developed at the inguinal region after orchiectomy, and define the sonography, color Doppler sonography and real-time ultrasound elastography findings in correlation with the histopathological findings.

## INTRODUCTION

A 25-year-old male with a history of left orchiectomy due to testis tumor eight months ago, presented with left inguinal palpable mass. The initial histopathological diagnosis was pT1 mixed germ cell tumor (80% yolk sac tumor and 20% embryonal carcinoma) which was 3cm in diameter, with necrosis but without vascular, rete, epididymis, spermatic cord, or tunica albuginea invasion. The surgical margins were free of tumor. Accompanying intratubular germ cell neoplasia was noted in the non-tumor testis tissue. The patient was then evaluated by thorax and abdomen CT which revealed three left para-aortic lymph nodes where the largest was 17mm in maximum transverse diameter. The patient was treated with two cycles of BEP (Bleomycin, Etoposide, Platinum) regimen, the chemotherapy was completed without any complications and he was taken into regular follow-up. At the time of diagnosis of the tumor, alpha-fetoprotein level was 51.5ng/mL (normal levels 0-7ng/mL) and ß-hCG level was 136.1ng/ mL (normal levels 0-5ng/mL) which came into the normal limits after surgery and chemotherapy.

**Figure 1:**
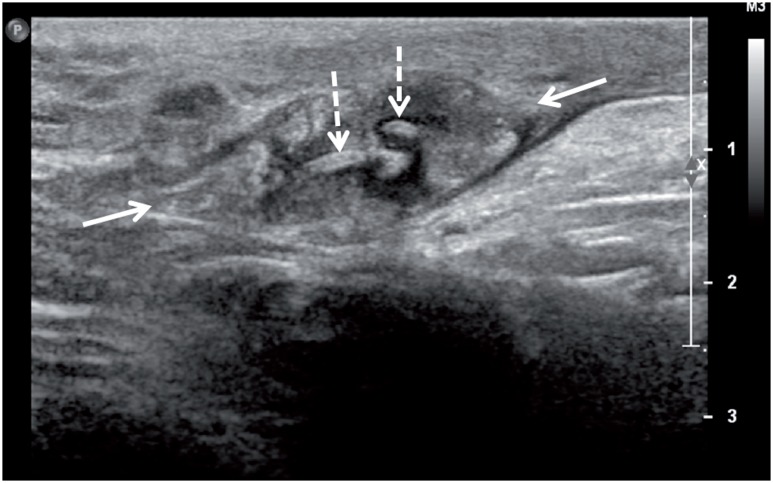
Gray-scale sonography of suture granuloma (arrows). It is observed centrally located curvilinear double echogenicity corresponding to suture material (dotted arrows) to be covered by highly sound attenuating tissue.

**Figure 2:**
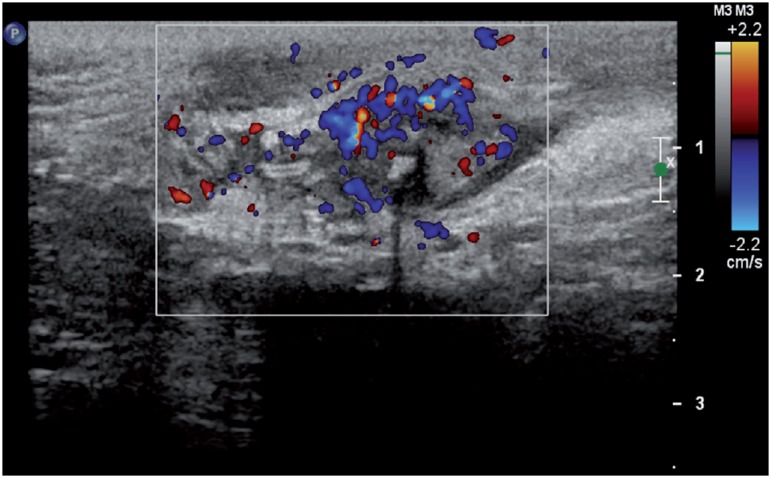
Color Doppler sonography of the granuloma shows the hypervascularity of the lesion.

**Figure 3:**
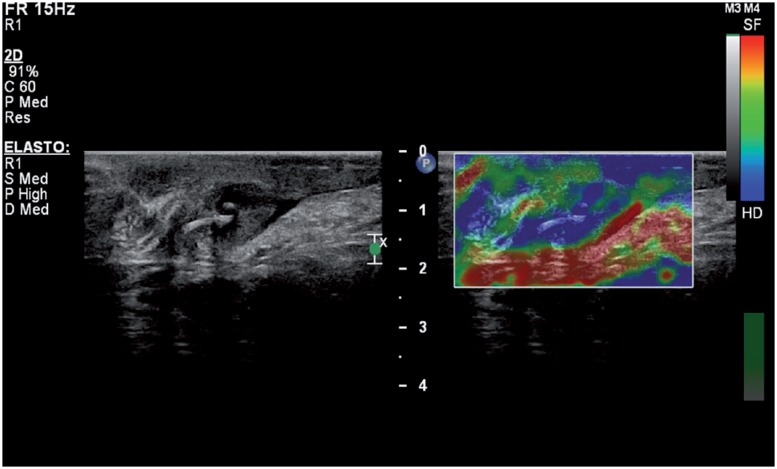
Real time ultrasound elastography demonstrates that the lesion is harder than the surrounding tissues.

**Figure 4:**
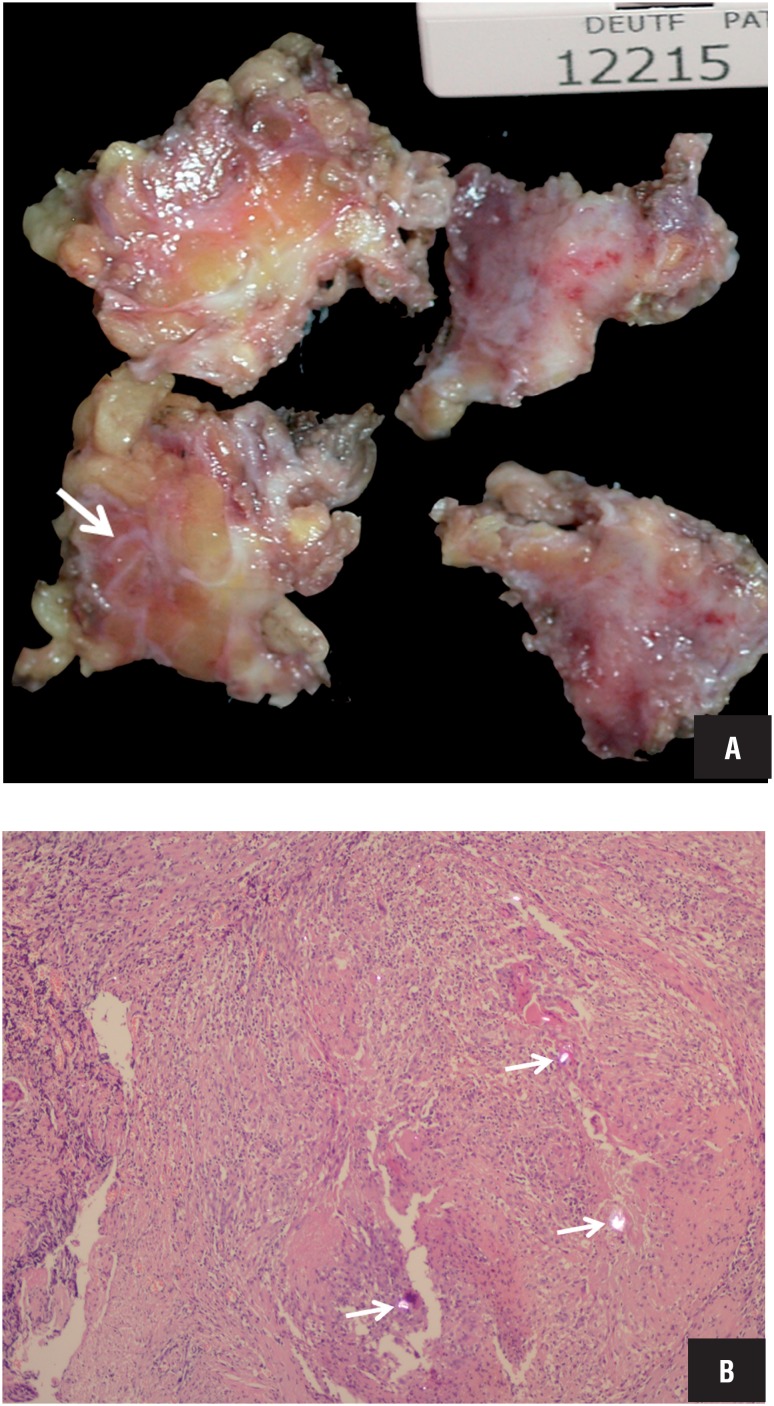
A) Macroscopy of the resected material. Arrow denotes the curvilinear appearance of silk and the reactive tissue around. B) Foreign material under polarized light (arrows) in a background of granulomatous reaction (H&E, x200).

Eight months after the initial admittance, the patient presented with a palpable, firm, non-painful, nodular mass in the proximal part of orchiectomy area in the left groin. Serum markers were within normal limits (AFP=4.5ng/mL, ß-hCG <1.0ng/mL). The patient was evaluated with sonographic examination by L5-12 transducer of a Philips iU22 scanner (Philips Medical Systems, Best, The Netherlands). On gray-scale sonography, the mass at the operation area was in soft tissue echogenicity 13*21*33mm in dimensions with lobulated but well defined margins (Figure-1). The lesion was found to include curvilinear tram-like highly echogenic material at the center and parallel lower echo areas covering that echogenicity. On color and power Doppler imaging, the lesion was highly vascular (Figure-2). Real-time ultrasound elastography demonstrated that the lesion had increased stiffness when compared to the surrounding soft tissues of the inguinal region, with high strain ratios, measured between 6 and 8 (Figure-3). The patient was operated, the inguinal lesion was excisionally removed and the histopathological investigation revealed that the lesion was a suture granuloma (Figure-4).

## DISCUSSION

Suture granuloma is a rare complication of surgery developing secondary to the use of non-absorbable suture material (1–9). It is a foreign body type of granulomatous reaction to a non-crystalline substance that is being processed to be eliminated by multinucleated giant cell formations. The development of suture granulomas has been reported to be a two-step process that begins with the initial reaction of tissue inflicted by the passage of the needle and then followed by a specific inflammatory reaction to the suture material (10).

Suture granulomas may occur anywhere in the body. The risk of development has been reported more with silk and polyfilament threads than the absorbable suture materials (3, 5). Silk is a braided suture material composed of protein fibers which is considered as a non-absorbable material. It is preferred because it is cheaper, more easily sterilized, tends to react less with the tissues and provides stronger wound closure (11). Inflammatory reaction to silk may sometimes be observed, even causing suppuration and sinus formation in wounds, but delayed inflammation of silk is known to be rare (2, 6, 11). However, a real epidemiological data is not existent.

Clinical presentation of suture granuloma may vary from a classic inflammatory reaction with erythema, swelling, pain and finally rejection of the suture material to a chronic inflammatory reaction with granuloma formation that may present as a solid mass, usually painless and gradually increasing in volume (12). The time duration between the operation and the occurrence of the granulomatous mass has been reported to vary between a few months to several years (2, 12).

The diagnosis of suture granuloma is particularly important in patients treated for cancer because the condition may resemble local tumor recurrence. Clinical findings, history and even the imaging features may be misleading. There are reported confusing lesions in the literature mostly detected by PET-CT (5–6). Suture granulomas mimicking intra-abdominal tumors on CT imaging have also been reported (7–9). Sonography, on the other hand, is probably the most helpful tool for the diagnosis of suture granulomas. Especially for superficially located lesions, high resolution sonographic imaging can easily detect the inside details of the granulomatous lesion as well the suture itself. Rettenbacher et al. (1) have reported the sonographic appearance indicating the pre-operative diagnosis of suture granuloma as hyperechogenic single or double lines inside of a hypoechogenic mass. Echogenicity has been attributed to the reflectivity of the suture itself, as supported by the sonographic bath experiment reported in the same article (1). In our patient, we observed curvilinear double layered echogenic material inside of the hypoechogenic soft tissue mass. In addition, we also detected that the echogenic suture material was covered by a prominent sound attenuating layer which was parallel to it. In pathological examination this layer was found to be the reactive tissue covering the suture material.

Color and power Doppler sonography exam in our patient showed that the lesion was hypervascular in nature particularly at the peripheral parts which were found to be inflammatory regions of the mass on histopathological evaluation. In fact, Doppler imaging had a limited role in the diagnosis of our case. Moreover, this hyper-vascular nature might have been misleading to a tumor diagnosis if the primary was a high stage tumor and/or the local recurrence an expected consequence.

We also performed real-time ultrasound elastography in our patient and investigated the strain ratio of the mass to evaluate its nature. Our case is the first one that elastography is used for the evaluation of a suture granuloma. Actually, elastography is a relatively new technique and the literature is limited at the moment in the field of characteristics of the granulomatous lesions (13, 14). However, it is not unexpected that the granulomatous lesions might show increased stiffness when compared to the surrounding soft tissue. In the presented case, the granulomatous lesion demonstrated prominent stiffness color, on color-scale and high values of 6 to 8 on strain ratio measurements.

The diagnosis of suture granuloma is provided by surgical excision of the lesion and histopathological examination. The foreign body is detectable at the center of the granuloma under polarized light, surrounded by multinucleated giant cells and macrophages. Surgery is not only used to achieve the final diagnosis but also used for eradication of the inflammation. In our patient, the local recurrence was not an expected condition because the local recurrence of stage pT1 testis tumor in a very short time after the completion chemotherapy, and with normal serology, is rare. Additionally, the sonographic findings were consistent with a suture granuloma. However, the patient was operated to confirm diagnosis, to clear up the lesional area and for the psychological relief of the patient.

In conclusion, suture granulomas are rare lesions that can be diagnosed by high resolution sonography with the typical features of single or double layer linear or curvilinear echogenicity covered by hypoechogenic layers. Doppler sonography seems to have a limited role to provide additional information for differential diagnosis. Elastography features reported here are the first in the literature and include increased stiffness with high strain ratios.
